# Attitudes toward Homosexuality and Same-Sex Marriage: The Roles of Parental Attitudes, Traditional Gender Role Values, and Filial Piety

**DOI:** 10.3390/ijerph20032194

**Published:** 2023-01-25

**Authors:** Ting Kin Ng, Ting Hin Lee, Hazyle Yuen, Wai Chan

**Affiliations:** 1 Department of Psychology, Lingnan University, 8 Castle Peak Road, Tuen Mun, New Territories, Hong Kong; 2 Department of Rehabilitation Sciences, The Hong Kong Polytechnic University, Hung Hom, Kowloon, Hong Kong

**Keywords:** attitudes toward homosexuality, attitudes toward same-sex marriage, parental attitudes, traditional gender role values, filial piety

## Abstract

Past studies have suggested that people’s attitudes toward homosexuality and same-sex marriages are influenced by their parents’ attitudes toward homosexuality. The current study intends to contribute to a more nuanced understanding of these associations by proposing a moderated mediation model incorporating traditional gender role values as a mediator and filial piety as a moderator. One hundred and fifteen adults (33.9% male and 66.1% female) aged from 18 to 36 years (M = 21.47, SD = 3.78) from Hong Kong completed an online questionnaire. The results of the latent moderated structural equations model showed that filial piety significantly moderated the indirect effects of negative parental attitudes toward homosexuality on attitudes toward homosexuality and attitudes toward same-sex marriage via traditional gender role values. The indirect effects were only significant when filial piety was high or medium but not when filial piety was low. These findings unpack the mechanisms underlying the effects of negative parental attitudes toward homosexuality on attitudes toward homosexuality and same-sex marriage and provide the boundary condition for the indirect effects of negative parental attitudes toward homosexuality on attitudes toward homosexuality and same-sex marriage through traditional gender role values.

## 1. Introduction

In recent decades, public attitudes toward homosexuality have become increasingly favorable worldwide [1]. However, many individuals still hold negative attitudes toward lesbian, gay, and bisexual people [2,3]. Homosexuals are prone to discrimination, rejection and violence in daily life owing to their sexual orientation [4]. Sexual orientation-based discrimination and violence are detrimental to the psychological well-being of homosexual individuals [5,6].

Researchers have long been interested in investigating factors that influence attitudes toward homosexuality [7]. More recently, some scholars have contended that although attitudes toward homosexuality appear to be directly related to attitudes toward same-sex marriage, the relationship between the two may be more complicated than it seems [8,9]. It has been argued that some people who personally oppose homosexuality may view same-sex marriage as a basic civil right for homosexuals [8]. Moreover, some opponents of same-sex marriage deny their disapproval of homosexuality and frame their rejection of same-sex marriage in terms of values such as tradition, democracy, and children’s welfare [9]. Therefore, this study focused on attitudes toward both homosexuality and sex-same marriage. The main aim of this study is to advance the extant literature by examining the effects of negative parental attitudes toward homosexuality on attitudes toward homosexuality and same-sex marriage, the mediating role of traditional gender role values, and the moderating role of filial piety among adults in Hong Kong.

### 1.1. Parental Attitudes toward Homosexuality

Parental attitudes toward homosexuality are crucial predictors of attitudes toward homosexuality and same-sex marriage. According to socialization theories, parents play critical roles in transmitting values, beliefs, traditions, and attitudes to their children [10]. Past studies have found that parental attitudes are predictors of children’s attitudes in general [11]. Parents often hold more negative attitudes toward homosexuality than their children do, as older generations tend to be less accepting of homosexuals compared with younger generations [12]. Research has found that parental disapproval of homosexuality is associated with more negative attitudes toward lesbian, gay, bisexual and transgender individuals [13] and same-sex marriage [14]. The impacts of parental attitudes may be especially pronounced in collectivistic Asian cultures, which emphasize family hierarchy [15,16] and parents’ responsibilities in educating their children [17,18]. Therefore, it is expected that negative parental attitudes toward homosexuality will be related to less positive attitudes toward homosexuality and same-sex marriage among adults in Hong Kong.

### 1.2. The Mediating Role of Traditional Gender Role Values

Although prior work has documented the effects of parental attitudes toward homosexuality on attitudes toward gays, lesbians and same-sex marriage [13,14], the mechanisms through which parental attitudes toward homosexuality influence attitudes toward homosexuality and same-sex marriage have not been well understood. The current study endeavors to unpack the indirect processes underlying these associations by examining the potential mediating role of traditional gender role values.

Traditional gender role values refer to social expectations that men and women should behave according to gendered stereotypes [19]. These values involve the beliefs that males should be masculine (e.g., independent, assertive, dominant) and females should be feminine (e.g., affectionate, caring, nurturance) [20]. Individuals who endorse traditional gender role values are likely to oppose homosexuality [21]. The traditional view of gender roles assumes that each individual should date or marry an opposite sex partner [22]. Therefore, same-sex relationships and marriage are regarded as violations of conventional gender roles [21,23]. Previous studies have affirmed that stronger endorsement of traditional gender role values is related to more negative attitudes toward homosexuality [21], and people with non-traditional gender role beliefs exhibited less rejection of lesbians and gays [24].

Parental attitudes toward homosexuality may influence endorsement of traditional gender values. As aforementioned, socialization theories highlight the key roles of parents in socializing values and attitudes to their children [10]. Apart from transferring anti-gay and anti-lesbian attitudes to their children, parents with negative attitudes toward homosexuality are likely to communicate traditional gender role values to their children and raise their children according to conventional gender stereotypes. It has been found that homophobic parents endorse traditional sex role stereotypes to a greater extent compared with non-homophobic parents [25]. Furthermore, research has also shown that more negative attitudes toward gays and lesbians are associated with more traditional gender role beliefs in child rearing [26]. Taken together, it is logical to predict that negative parental attitudes toward homosexuality will be associated with higher endorsement of traditional gender role values, which in turn will be associated with less accepting attitudes toward homosexuality and same-sex marriage.

### 1.3. The Moderating Role of Filial Piety

If people’s attitudes toward homosexuality mainly depend on their parents’ attitudes, it would be surprising that younger generations hold more favorable attitudes toward homosexuality compared with older generations [12]. Research on parental attitudes toward homosexuality has yielded mixed evidence. For instance, a study found that college students’ attitudes toward homosexuality were not significantly related to negative parental attitudes toward homosexuality [27]. The inconsistent findings suggest that the relationship may be influenced by a moderator [28,29].

This study seeks to extend the existing literature by investigating the potential moderating role of filial piety in the relationships among parental attitudes toward homosexuality, traditional gender role values, and attitudes toward homosexuality and same-sex marriage. Filial piety refers to a set of expectations of children’s behavior toward their parents, including obedience, respect, and providing care and financial support to aged parents [27,30]. Filial piety is linked to disapproval of homosexuality, because establishing a traditional family and preserving the continuity of the family bloodline are regarded as crucial filial obligations [27]. Past studies have revealed a negative relationship between filial piety and attitudes toward homosexuality [27,31].

Filial piety may enhance the effect of parental attitudes on children’s attitudes. As obeying and respecting parents are core filial piety values [27], children who are more filial tend to have greater willingness to be socialized by their parents [32]. One study showed that filial piety significantly moderated the relationship between parental attitudes toward marriage and negative lesbian, gay, or bisexual identities among lesbian, gay, or bisexual students, such that the relationship was only significant when filial piety was high but not when filial piety was low [31]. In this light, filial piety may provide a boundary condition for the direct effects of negative parental attitudes toward homosexuality on traditional gender role values and attitudes toward homosexuality and same-sex marriage, as well as the indirect effect of negative parental attitudes toward homosexuality on attitudes toward homosexuality and same-sex marriage through traditional gender role values.

### 1.4. Potential Effects of Gender, Age, and Sexual Orientation

Past studies have suggested that gender, age, and sexual orientation may influence attitudes toward homosexuality and same-sex marriage. One study revealed that men held more negative attitudes toward gay marriage and lesbian marriage than women did [33]. Moreover, younger people tended to have more favorable attitudes toward homosexuals compared with older people [34]. Furthermore, straight men were found to hold more negative attitudes toward homosexuality than gay men did [35]. Therefore, this study included gender, age, and sexual orientation as control variables.

### 1.5. The Current Study

**Figure 1:**
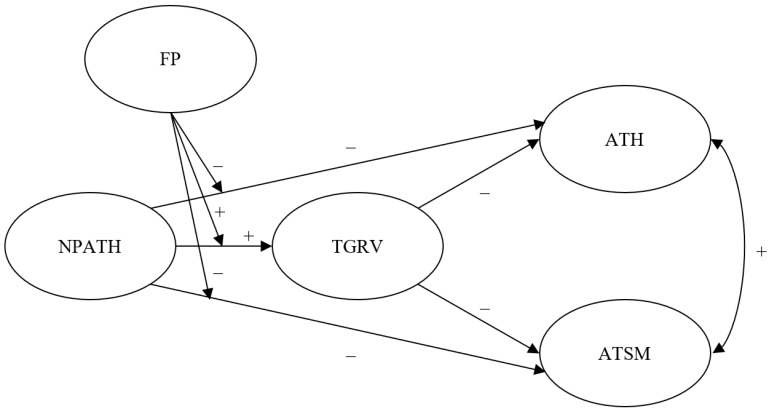
Hypothesized moderated mediation model. NPATH = negative parental attitudes toward homosexuality; FP = filial piety; TGRV = traditional gender role values; ATH = attitudes toward homosexuality; ATSM = attitudes toward same-sex marriage.

Prior studies have predominantly focused on the direct effects of parental attitudes toward homosexuality on attitudes toward homosexuality and same-sex marriage [12,13]. The present study aims to contribute to a more comprehensive understanding of these relationships by investigating the mediating effect of traditional gender role values and the moderating effect of filial piety among adults in Hong Kong. The proposed moderated mediation model is illustrated in Figure 1. Several hypotheses were proposed. First, it is hypothesized that the associations between negative parental attitudes toward homosexuality and attitudes toward homosexuality and same-sex marriage will be mediated by traditional gender role values. Second, it is hypothesized that filial piety will moderate the effects of negative parental attitudes toward homosexuality on traditional gender role values and attitudes toward homosexuality and same-sex marriage, such that the effects of negative parental attitudes toward homosexuality will be stronger when filial piety is higher. Third, it is hypothesized that filial piety will moderate the indirect effect of negative parental attitudes toward homosexuality on attitudes toward homosexuality and same-sex marriage via traditional gender role values, such that the indirect effect will be stronger when filial piety is higher.

## 2. Method

### 2.1. Participants and Procedure

One hundred and fifteen adults from Hong Kong participated in this study. Among them, 33.9% were male and 66.1% were female. Their age ranged from 18 to 36 years (M = 21.47, SD = 3.78). With respect to their sexual orientation, 81.7% were heterosexual, 7.8% were homosexual, and 10.4% were bisexual.

Data were collected using an online questionnaire written in English. Participants were recruited either through the participation pool of an Introduction to Psychology course (74.8%) or through personal contacts (25.2%). Participation was voluntary and anonymous. On average, the questionnaire took about 15 min to complete.

### 2.2. Measures

#### 2.2.1. Attitudes toward Homosexuality

Participants’ attitudes toward homosexuality were measured using the Attitudes Toward Homosexuality Scale (ATHS) [2,36]. The original ATHS consists of 25 items written in French [36]. This study adopted the 16-item English version of the ATHS [2]. A sample item is “Homosexuality is a natural expression of affection and sexuality”. Respondents were asked to report to the extent to which they agreed upon each statement on a 7-point scale ranging from 1 (strongly disagree) to 7 (strongly agree). A higher scale score represents more positive attitudes toward homosexuality.

#### 2.2.2. Attitudes toward Same-Sex Marriage

The Attitudes Toward Same-Sex Marriage Scale (ATSM) [8] was employed to assess participants’ attitudes toward same-sex marriage. A sample item is “Same-sex marriage ensures equal rights for all relationships regardless of sexual orientation”. Respondents were asked to indicate the degree to which they agreed upon each statement on a 5-point scale ranging from 1 (strongly disagree) to 5 (strongly agree). A higher score indicates more positive attitudes toward homosexual marriage.

#### 2.2.3. Negative Parental Attitudes toward Homosexuality

Negative parental attitudes toward homosexuality were measured using a two-item measure [27]. A sample item is “My parents disapprove of homosexuality”. The items were rated on a 5-point scale ranging from 1 (strongly disagree) to 5 (strongly agree). A higher score reflects more negative parental attitudes toward homosexuality.

#### 2.2.4. Filial Piety

Participants’ level of endorsement of filial piety was assessed with a four-item measure [27]. One sample item is “It is important for me to respect my parents”. Each item was scored on a 4-point scale ranging from 1 (strongly disagree) to 4 (strongly agree). A higher score represents a greater endorsement of filial piety.

#### 2.2.5. Traditional Gender Role Values

Participants’ level of endorsement of traditional gender role values was measured by a four-item measure [21]. One sample item is “When jobs are scarce, men should have more right to a job than women”. All items were rated on a 4-point scale ranging from 1 (strongly disagree) to 4 (strongly agree). A higher scale score indicates greater endorsement of traditional gender role values.

## 3. Data Analysis

Considering the small sample size relative to the number of measurement items, three item categories were constructed for each latent construct [37], apart from the two-item measure of negative parental attitudes toward homosexuality. To test the hypothesized moderated mediating effects, the latent moderated structural equations (LMS) method [38,39] with maximum likelihood estimation was performed using Mplus. In the first step, a model without the interaction term (Model 0) was analyzed. Next, the latent interaction term (negative parental attitudes toward homosexuality × filial piety) was generated using the XWITH function of Mplus [39], and LMS models with the effects of the latent interaction term on attitudes toward homosexuality (Model 1a), attitudes toward same-sex marriage (Model 1b), and traditional gender role values (Model 1c) were analyzed. The potential effects of gender, age, and sexual orientation on the dependent variables (attitudes toward homosexuality and attitudes toward same-sex marriage) and mediator (traditional gender role values) were controlled for in all models. Gender was coded as a dummy variable (1 = female, 0 = male). Sexual orientation was represented by two dummy variables, homosexual orientation (1 = yes, 0 = no) and bisexual orientation (1 = yes, 0 = no) and heterosexual orientation served as the reference category.

The goodness-of-fit of Model 0 was evaluated using a combination of fit statistics, including the root mean square error of approximation (RMSEA), and standardized root mean square residual (SRMR), the comparative fit index (CFI), and the Tucker–Lewis index (TLI). A good model fit is indicated by a RMSEA < 0.06, a SRMR < 0.08, a CFI > 0.95, and a TLI > 0.95 [40]. Fit statistics cannot be estimated when the XWITH function of Mplus is applied to analyze a LMS model [39]. Therefore, a log-likelihood test was conducted to compare Model 0 with Model 1a, 1b and 1c. A significant result suggests that the inclusion of the interaction effect significantly improves the model fit. If Model 0 showed a good fit and the log-likelihood test was significant, the LMS model (Model 1a, 1b or 1c) also fitted the data well [38,39]. Hypothesized effects were examined using one-tailed tests. The predicted moderated mediating effects and conditional indirect effects were analyzed using the bootstrapping technique, which has advantages over other indirect effect tests with lower statistical power and reliance on the normality assumption [41]. Biased-corrected 90% confidence intervals (BC 90% CIs) were generated with 1000 resamples as one-tailed tests of moderated mediating effects and conditional indirect effects at the 0.05 confidence level.

## 4. Results

### 4.1. Descriptive Statistics and Preliminary Analyses

The descriptive statistics of the study variables are listed in Table 1. All variables demonstrated satisfactory internal consistency reliability (α = 0.78 to 0.91). Attitudes toward same-sex marriage were positively associated with attitudes toward homosexuality and negatively associated with negative parental attitudes toward homosexuality, filial piety, and traditional gender role values. Attitudes toward homosexuality were negatively associated with negative parental attitudes toward homosexuality and traditional gender role values. Traditional gender role values were positively associated with negative parental attitudes toward homosexuality and filial piety.

Table 2 shows the demographic and study variables by gender. Males reported higher endorsement of filial piety and traditional gender role values than females did. No gender differences were observed for other variables.

### 4.2. Latent Moderated Structural Equations

The model without the interaction term (Model 0) achieved an excellent model fit, χ^2^(138) = 174.65, *p* = 0.019, RMSEA = 0.048, 90% CI [0.021, 0.069], SRMR = 0.055, CFI = 0.97, TLI = 0.96. The standardized factor loadings ranged from 0.66 to 0.94. Compared to Model 0, the model with the negative parental attitudes toward homosexuality × filial piety interaction effect on attitudes toward homosexuality (Model 1a) did not fit the data better, χ^2^(1) = 0.02, *p* = 0.888. The model with the negative parental attitudes toward homosexuality × filial piety interaction effect on attitudes toward same-sex marriage (Model 1b) was also not significantly superior to Model 0, χ^2^(1) = 0.41, *p* = 0.523. These two interaction effects were not added.

**Figure 2:**
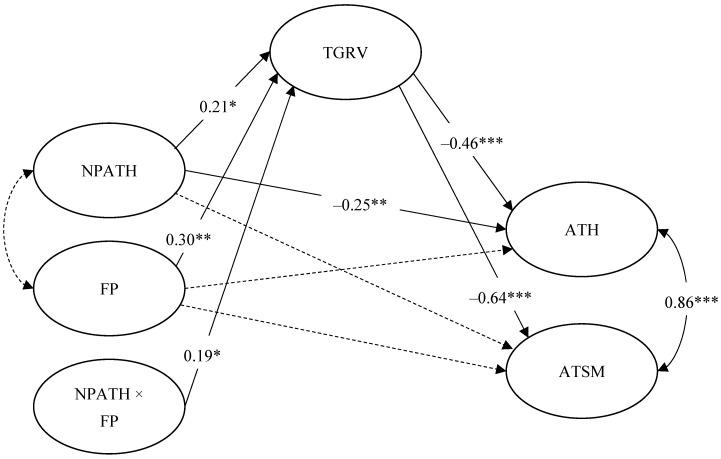
Final moderated mediation model. Solid lines represent significant paths. Dashed lines represent non-significant paths. Significant standardized coefficients are presented. Control variables (gender, age, and sexual orientation), observed indicators and error variances are omitted for clarity. NPATH = negative parental attitudes toward homosexuality; FP = filial piety; TGRV = traditional gender role values; ATH = attitudes toward homosexuality; ATSM = attitudes toward same-sex marriage. * *p* < 0.05. ** *p* < 0.01. *** *p* < 0.001.

The model with the negative parental attitudes toward homosexuality × filial piety interaction effect on traditional gender role values (Model 1c) was significantly better than Model 0, χ^2^(1) = 3.86, *p* = 0.049, indicating that the addition of the interaction effect resulted in improved model fit. Model 1c was retained as the final model (see Figure 2).

Regarding the effects of control variables, traditional gender role values were positively predicted by age (β = 0.27, *p* = 0.002) and negatively predicted by female gender (β = −0.31, *p* < 0.001). Moreover, attitudes toward homosexuality were positively predicted by homosexual orientation (β = 0.37, *p* < 0.001) and bisexual orientation (β = 0.35, *p* < 0.001). Furthermore, attitudes toward same-sex marriage were negatively predicted by female gender (β = −0.19, *p* = 0.010) and positively predicted by homosexual orientation (β = 0.15, *p* = 0.027) and bisexual orientation (β = 0.30, *p* < 0.001).

Attitudes toward homosexuality were negatively predicted by traditional gender role values (β = −0.46, *p* < 0.001) and negative parental attitudes toward homosexuality (β = −0.25, *p* = 0.004). Attitudes toward same-sex marriage were negatively predicted by traditional gender role values (β = −0.64, *p* < 0.001). Traditional gender role values were negatively predicted by negative parental attitudes toward homosexuality and (β = 0.21, *p* = 0.013) filial piety (β = 0.30, *p* = 0.002).

**Figure 3:**
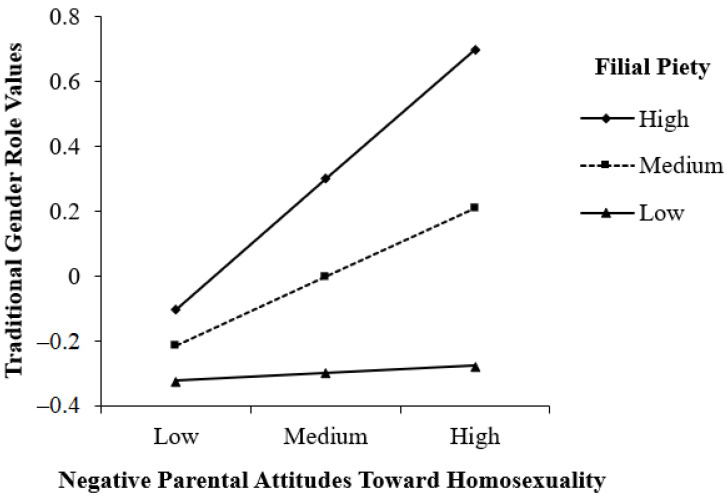
The simple main effect of negative parental attitudes toward homosexuality on traditional gender role value at high (M + 1SD), medium (M), and low (M – 1SD) levels of filial piety.

Moreover, filial piety significantly enhanced the effect of negative parental attitudes toward homosexuality on traditional gender role values (β = 0.19, *p* = 0.025). Simple slopes were investigated at high (M + 1SD), medium (M), and low (M – 1SD) levels of filial piety (see Figure 3). The simple main effect of negative parental attitudes toward homosexuality on traditional gender role values was significant when filial piety was high (β = 0.40, *p* = 0.003) or medium (β = 0.21, *p* = 0.013), but not when filial piety was low (β = 0.02, *p* = 0.433).

The results of moderated mediation analyses are summarized in Table 3. Filial piety significantly enhanced the negative indirect effect of negative parental attitudes toward homosexuality on attitudes toward homosexuality via traditional gender role values (index of moderated mediation = −0.19, BC 90% CI [−0.47, −0.02]). The indirect effect was significant at high or medium filial piety but not at low filial piety (see Table 2). Furthermore, filial piety significantly strengthened the negative indirect effect of negative parental attitudes toward homosexuality on attitudes toward same-sex marriage through traditional gender role values (index of moderated mediation = −0.20, BC 90% CI [−0.49, −0.02]). The indirect effect was significant at high or medium filial piety but not at low filial piety (see Table 2).

## 5. Discussion

Previous research has linked parental attitudes toward homosexuality to attitudes toward gays, lesbians and same-sex marriage [12,13]. The current study attempts to elaborate on these relationships by testing a moderated mediation model, in which traditional gender role values served as a mediator and filial piety functioned as a moderator. In line with our hypothesis, this study showed that filial piety significantly enhanced the effect of negative parental attitudes toward homosexuality on traditional gender role values. Furthermore, it was also shown that filial piety significantly enhanced the indirect effects of negative parental attitudes toward homosexuality on attitudes toward homosexuality and same-sex marriage via traditional gender role values.

This study found that negative parental attitudes toward homosexuality were associated with greater endorsement of traditional gender role values. This finding echoes the previous research findings that more homophobic parents have a greater endorsement of traditional sex roles [25], and negative attitudes toward gays and lesbians are related to more traditional gender role beliefs in child rearing [26]. Furthermore, this study revealed that filial piety significantly enhanced the association between negative parental attitudes toward homosexuality and traditional gender role values. Negative parental attitudes toward homosexuality were associated with traditional gender role values only for those with high or medium levels of filial piety, but not for those with a low level of filial piety. This finding is in accordance with the notion that more filial children may be more willing to internalize values socialized by their parents [32]. Nonetheless, filial piety did not significantly moderate the direct effects of negative parental attitudes toward homosexuality on attitudes toward homosexuality and same-sex marriage. It may be because the effects of negative parental attitudes toward homosexuality were mediated by traditional gender role values.

More importantly, the current results indicated that filial piety significantly exacerbated the indirect effects of negative parental attitudes toward homosexuality on attitudes toward homosexuality and same-sex marriage via traditional gender role values. One study found that filial piety significantly enhanced the effect of parental attitudes toward marriage on the negative lesbian, gay and bisexual identities of homosexual and bisexual students, such that the effect of parental attitudes toward marriage was only significant at a high level of filial piety [31]. The current study further identified filial piety as the boundary condition for the indirect effects of negative parental attitudes toward homosexuality on attitudes toward homosexuality and same-sex marriage via traditional gender role values. For adults with high or medium levels of filial piety, negative parental attitudes toward homosexuality were positively related to greater endorsement of traditional gender role values, which in turn was negatively related to attitudes toward homosexuality and same-sex marriage. However, these indirect effects were not observed for adults with a low level of filial piety. This finding shed light on the mechanisms through which parental attitudes toward homosexuality influence attitudes toward homosexuality and same-sex marriage. Furthermore, it was found that negative parental attitudes toward homosexuality had a significant direct effect on attitudes toward homosexuality but not on attitudes toward same-sex marriage after controlling for traditional gender role values. One possible explanation may be that opposite-sex marriage is one major aspect of conventional gender roles [22]. Thus, the effect of negative parental attitudes toward homosexuality on attitudes toward same-sex marriage may be completely mediated by traditional gender role values. Further investigations are required to verify this claim.

### 5.1. Implications

The findings of this study have important practical implications. In particular, the present findings indicate that more negative parental attitudes toward homosexuality lead to less accepting attitudes toward homosexuality via traditional gender role values, especially for those who have high or medium levels of filial piety. In this light, interventions that aim at promoting tolerance for homosexuality will not only influence parents’ attitudes but also their children’s attitudes. Research has supported the effectiveness of education programs about homosexuality in promoting more tolerant attitudes toward homosexuality [42]. Future studies are recommended to investigate whether delivering education programs about homosexuality to parents will also change their children’s attitudes toward homosexuality.

### 5.2. Limitations and Directions for Future Research

In spite of its theoretical contributions, this study was not without limitations. First, this study adopted a cross-sectional survey design, which precludes inferences about the causal directions among the study variables [43]. Future work is suggested to employ a longitudinal design to provide stronger evidence for the causality among variables. Second, this study used self-report measures of attitudes toward homosexuality and same-sex marriage. Self-report measures are prone to social desirability bias [44], especially questions on sensitive topics such as attitudes toward homosexuality [45]. Further research is recommended to adopt an implicit measure of attitudes toward homosexuality [46] in addition to an explicit self-report measure. Third, the sample of this study only included adults. Researchers have argued that parenting may be more influential for children and adolescents than for adults [47]. Further studies are needed to examine the effects of parental attitudes on traditional gender role values and attitudes toward homosexuality and same-sex marriage among children and adolescents. Fourth, this study only recruited participants from Hong Kong. Scholars have noted that filial piety [48] and parents’ obligations in disciplining their children [17,18] are highly valued in collectivistic Asian cultures. Future research is required to verify the roles of parental attitudes and filial piety on traditional gender role values and attitudes toward homosexuality and same-sex marriage among individuals from other cultures. Fifth, most participants in this study were heterosexual. As shown in the current results, homosexual and bisexual participants reported more positive attitudes toward homosexuality and same-sex marriage compared with heterosexual participants. Future research is suggested to further examine the observed moderated mediating effects among homosexual and bisexual individuals.

## 6. Conclusions

The present study attempts to offer a fuller understanding of the associations of parental attitudes toward homosexuality with attitudes toward homosexuality and same-sex marriage. We proposed a moderated mediation model in which traditional gender role values served as a mediator and filial piety served as a moderator. The results of the LMS model revealed that the indirect effects of negative parental attitudes toward homosexuality on attitudes toward homosexuality and attitudes toward same-sex marriage through traditional gender role values were significantly moderated by filial piety. The indirect effects emerged at high or medium levels of filial piety, but not at a low level of filial piety. The current findings disentangle the mechanisms through which negative parental attitudes toward homosexuality influence attitudes toward homosexuality and same-sex marriage and identify the boundary condition for the indirect processes.

## Figures and Tables

**Table 1:** Descriptive Statistics of the Study Variables.

	M	SD	1	2	3	4	5
1. NPATH	3.31	1.02	(0.79)				
2. FP	2.83	0.66	0.10	(0.78)			
3. TGRV	1.60	0.71	0.16 *	0.25 **	(0.89)		
4. ATH	5.14	0.97	−0.26 **	−0.15	−0.42 ***	(0.89)	
5. ATSM	3.90	0.69	−0.23 **	−0.29 **	−0.52 ***	0.81 ***	(0.91)

Note. NPATH = negative parental attitudes toward homosexuality; FP = filial piety; TGRV = traditional gender role values; ATH = attitudes toward homosexuality; ATSM = attitudes toward same-sex marriage. Values on the diagonal are Cronbach’s α coefficients. * *p* < 0.05. ** *p* < 0.01. *** *p* < 0.001.

**Table 2:** Demographic and Study Variables by Gender.

	Male		Female		
	*n*	%	*n*	%	χ^2^
Sexual orientation					1.19
Heterosexual	34	87.2	60	78.9	
Homosexual	2	5.1	7	9.2	
Bisexual	3	7.7	9	11.8	
	M	SD	M	SD	t
Age	21.38	3.66	21.51	3.87	−0.17
NPATH	3.23	0.94	3.35	1.07	−0.58
FP	2.98	0.63	2.76	0.67	1.70 *
TGRV	1.94	0.76	1.43	0.61	3.93 ***
ATH	4.96	0.91	5.24	1.0	−1.47
ATSM	3.84	0.67	3.92	0.70	−0.62

Note. NPATH = negative parental attitudes toward homosexuality; FP = filial piety; TGRV = traditional gender role values; ATH = attitudes toward homosexuality; ATSM = attitudes toward same-sex marriage. * *p* < 0.05. *** *p* < 0.001.

**Table 3:** Indices of Moderated Mediation and Conditional Indirect Effects.

	*b*	BC 90% CI	β
Indirect effect: NPATH → TGRV → ATH			
Low FP	−0.01	[−0.14, 0.10]	−0.01
Medium FP	−0.12 *	[−0.27, −0.04]	−0.10
High FP	−0.22 *	[−0.48, −0.07]	−0.18
Index of moderated mediation	−0.19 *	[−0.47, −0.02]	−0.09
Indirect effect: NPATH → TGRV → ATSM			
Low FP	−0.01	[−0.14, 0.10]	−0.01
Medium FP	−0.13 *	[−0.26, −0.04]	−0.14
High FP	−0.24 *	[−0.48, −0.08]	−0.26
Index of moderated mediation	−0.20 *	[−0.49, −0.02]	−0.12

Note. NPATH = negative parental attitudes toward homosexuality; FP = filial piety; TGRV = traditional gender role values; ATH = attitudes toward homosexuality; ATSM = attitudes toward same-sex marriage. BC 90% CI = bias-corrected 90% confidence interval. * *p* < 0.05.

## Data Availability

The data presented in this study are available upon reasonable request from the corresponding author.
